# Essential oil of *Melaleuca alternifolia* for the treatment of oral candidiasis induced in an immunosuppressed mouse model

**DOI:** 10.1186/1472-6882-14-489

**Published:** 2014-12-15

**Authors:** Vanessa Maria de Campos Rasteiro, Anna Carolina Borges Pereira da Costa, Cássia Fernandes Araújo, Patrícia Pimentel de Barros, Rodnei Dennis Rossoni, Ana Lia Anbinder, Antonio Olavo Cardoso Jorge, Juliana Campos Junqueira

**Affiliations:** Department of Biosciences and Oral Diagnosis, Institute of Science and Technology, UNESP - Univ Estadual Paulista, Francisco José Longo 777, São Dimas, São José dos Campos CEP, Kragujevac, 12245-000 SP Brazil

**Keywords:** *Melaleuca alternifolia*, *Candida albicans*, Oral candidiasis, Murine model

## Abstract

**Background:**

The search for alternative therapies for oral candidiasis is a necessity and the use of medicinal plants seems to be one of the promising solutions. The objective of this study was to evaluate the *in vitro* and *in vivo* effects of the essential oil of *Melaleuca alternifolia* on *Candida albicans*.

**Methods:**

The minimum inhibitory concentration (MIC) and minimum biofilm eradication concentration (MBEC) of *M. alternifolia* were determined by the broth microdilution assay. For the *in vivo* study, twelve immunosuppressed mice with buccal candidiasis received topical applications of *M. alternifolia* with MBEC. After treatment, yeasts were recovered from the mice and quantified (CFU/mL). Mice were killed for morphologic analysis of the tongue dorsum by optical and scanning electron microscopy. Data were analyzed using Student’s *t* test or Mann-Whitney test.

**Results:**

The MIC of *M. alternifolia* was 0.195% and the MBEC was 12.5%. Treatment with *M. alternifolia* achieved a 5.33 log reduction in *C. albicans* and reduced the microscopic lesions of candidiasis.

**Conclusions:**

*M. alternifolia* oil at a 12.5% was effective to eradicate a *C. albicans* biofilm formed *in vitro* and to reduce yeasts of *C. albicans* in an immunosuppressed mouse model.

## Background

Recent decades have seen a significant increase in the incidence of all forms of candidiasis. This increase reflects changes in medical practice with more frequent use of invasive surgical procedures, and more widespread use of immunosuppressive therapies and broad-spectrum antibiotics [1]. However, key to the increase in oral candidiasis has been the expansion of acquired immunodeficiency syndrome (AIDS) worldwide [1]. Oral candidiasis is the most common fungal infection in patients with AIDS and it usually indicates the progression of HIV infection [2–4]. Treatment of oral candidiasis in HIV-positive patients is difficult because of recurring episodes, intermittent exposure and continual selection of antifungal therapy-resistant strains. Studies have shown that *C. albicans* and non-*albicans* strains are developing resistance to the antifungals widely used for treatment of oral candidiasis, such as fluconazole and amphotericin B [5, 6].

Another aspect related to antifungal resistance and the recurrence of infection is the ability of *Candida* spp. to form biofilms on various surfaces in the oral cavity. A biofilm has been defined as a community of microorganisms organized at interfaces, enclosed in a self-produced polymeric matrix and adhered to an inert or living tissue. The presence of an exopolymeric matrix couple with the organization of layers of cells may confer protection of organisms in the inner layers, thereby contributing to antifungal resistance [7–9]. For example, biofilms formed *in vitro* by *C. albicans* on silicone catheters were sensitive to micafungin only at a concentration 100 to 500 times higher than the minimum inhibitory concentration (MIC) in planktonic cells [10].

The search for alternative therapies for oral candidiasis is a necessity and the use of medicinal plants seems to be one of the promising solutions [11, 12]. The plant *Melaleuca alternifolia* has been used as an antiseptic remedy for decades. Although there is no published documentation of specific medicinal applications for *M. alternifolia* by Aboriginals prior to the colonization of Australia, the Bundjalung Aboriginals of New South Wales used the plant for medicinal purposes and spoke of the wound healing properties of the water from a lake into which *M. alternifolia* leaves had fallen [13]. The essential oil of *M. alternifolia*, termed tea tree oil, contains almost hundreds components, the majority of which are monoterpenes and related alcohols. It has a minimum content of 30% of terpinen-4-ol and a maximum content of 15% of 1,8-cineole. Terpinen-4-ol is a major *M. alternifolia* component and exhibits strong antimicrobial and anti-inflammatory properties, whereas 1,8-cineole is probably an undesirable allergen in *M. alternifolia* products [14]. Preliminary trials suggest that *M. alternifolia* formulations may be effective in the treatment of acne and fungal infections, and in bacterial pathogen decolonization protocols [13]. The antimicrobial activity of *M. alternifolia* is attributed to its ability to denature proteins and alter the properties and function of the cell wall membrane, leading to the loss of intracellular components and eventually cell death [15–17].

Several *in vitro* studies described the antifungal activity of *M. alternifolia* against *Candida* isolates in planktonic growth, with MICs typically between 0.5-2% (v/v) [17] and showed inhibition of germ tube formation by *C. albicans* [18]. Few studies were performed *in vivo* to confirm and strengthen the *in vitro* results [19, 20]. Because *M. alternifolia* shows promise as a topical anti-candidal therapy and its antimicrobial effects depend on the concentration used [17], the aim of this study was to determine the minimal concentration of *M. alternifolia* required to eradicate *C. albicans* biofilms formed *in vitro* and to study the effects of this concentration in treating oral candidiasis induced in an immunosuppressed mouse model.

## Methods

### Microbial strains and culture conditions

*C. albicans* strain ATCC 18804 was used for all experimental assays in this study. This strain was stored as frozen stocks in 30% glycerol at -80°C, subcultured on Sabouraud Dextrose agar plates and routinely grown in Sabouraud liquid medium at 37°C.

### *M. alternifolia*essential oil

*M. alternifolia* was purchased at the Federal University of Viçosa/UFV. The UFV has the botanical identification of the plant studied, as well as its own deposit in a herbarium voucher specimen of the University, with registration number 30839 corresponding to *M. alternifolia*. The oil was obtained by the steam distillation technique. The oil sample contained the following major components: terpinen-4-ol (42.8%), γ-terpinene (20.4%), *p*-cymene (9.6%), α-terpinene (7.9%), 1,8-cineole (3%), α-terpineol (2,8%) and α-pinene (2.4%) as determined by gas chromatography mass spectrometry [21].

### *In vitro*activity of *M. alternifolia*essential oil: Determination of Minimal Inhibitory Concentration (MIC) in planktonic culture

The MIC of *M. alternifolia* was determined by the broth microdilution method according to the Clinical and Laboratory Standards Institute (CLSI) [22] document M27-A2. Initially, *C. albicans* were grown in Sabouraud dextrose agar for 48 h at 37°C. Then, the yeast suspension was prepared in 5 mL sterile saline (0.85%) and the cellular density was adjusted to 0.284 using a spectrophotometer (B582, Micronal, São Paulo, Brazil) at wavelength = 530 nm, resulting in a standard solution with 1 × 10^6^ cells/mL. The standard solution was diluted 1:50 in RPMI 1640 medium with L-glutamine without sodium bicarbonate and buffered with 0.165 M morpholinepropanesulfonic acid (MOPS; Sigma-Aldrich, Steinheim, Germany), followed by a 1:20 dilution to obtain a final concentration of 1-5 × 10^3^ cells/mL.

Eleven serial 1:2 dilutions were made from the *M. alternifolia* into a 96-well plate (25 to 0.01% (v/v)) from 100 μL of *M. alternifolia* in 100 μL of culture medium RPMI 1640 at pH 7.0 ± 0.1. Subsequently, 100 μL of the standardized suspension of *C. albicans* were added to each well of 96-well plate. A well for positive control (medium with inoculum) and another well for negative control (medium alone) were included.

Tween-80 (final concentration 0.001% v/v) was included to facilitate oil solubility [19]. The plates were incubated at 37°C for 24 h. Minimal inhibitory concentration (MIC) was determined in the well of lowest concentration, in which turbidity was not observed when compared to oil-free growth control. This experiment was performed independently in triplicate.

### *In vitro*activity of *M. alternifolia*essential oil: Determination of Minimal Biofilm Eradication Concentration

*C. albicans* biofilms were formed *in vitro* using the methodology described by Seneviratne *et al*. [23] with some modifications. Cultures of *C. albicans* grown on Sabouraud dextrose agar (Himedia) at 37°C for 18h were harvested in yeast nitrogen base (YNB, Himedia) supplemented with 50 mM glucose (Vetec, Duque de Caxias, RJ, Brazil). After an 18 h incubation at 37°C , yeasts were centrifuged at 358 × *g* for 10 minutes, washed twice with PBS, resuspended with YNB supplemented with 100 mM glucose and adjusted to an optical density of 0.381 at 530 nm (10^7^ cells/mL) using a spectrophotometer (B582, Micronal). A 250 μL aliquot of *C. albicans* suspension was pipetted into each well of a 96-well flat-bottom microtiter plate (Costa Corning). The plate was incubated for 90 min at 37°C in a shaker at 75 rpm (Quimis, Diadema, Brazil) for the initial adhesion phase. After this period, the wells were washed with 250 μL of PBS to removed loosely adherent cells. A 250 μL aliquot of YNB supplemented with 100 mM glucose was then pipetted into each washed well, and the plates were incubated at 37°C in a shaker at 75 rpm for 48 h. The broth was changed every 24 h. Plates with biofilms formed by *C. albicans* were washed with 250 μL of PBS to remove loosely adherent cells.

The biofilm formed in each well was immersed in 250 μL of *M. alternifolia* suspended in 1% Tween 80 for 5 min in an orbital shaker (Solab, Piracibaca, Brazil). The concentration of *M. alternifolia* tested ranged from the MIC to 25%. The biofilm of the control group was pipetted with phosphate buffered saline (PBS) for the same period of time. All tests were performed in triplicate.

After treatments with *M. alternifolia*, biofilm cells were scraped off the well wall using a sterile toothpick and transferred to Falcon tubes containing 10 mL PBS. To disrupt the biofilms, the contents of the tubes were homogenized for 30 s using an ultrasonic homogenizer (Sonoplus HD 2200, Badelin Electronic, Berlim, Germany) with an output power of 50 W. The solution in the Falcon tubes was considered to be diluted by a factor of 10^-1^. Serial dilutions were then made using each original 10^-1^ dilution, and aliquots of 0.1mL were seeded onto Sabouraud dextrose agar (Himedia) plates that were incubated at 37°C for 48 h. After the incubation period, CFU/mL values were determined for each plate. Minimal biofilm eradication concentration was defined as the lowest concentration of oil that resulted in complete inhibition of CFU/mL.

### Analysis of *C. albicans*biofilms formed *in vitro*by Scanning Electron Microscopy (SEM)

Biofilms of *C. albicans* treated with the minimal biofilm eradication concentration of TTO (n = 2) and treated with PBS as a control group (n = 2) were subjected to SEM analysis.

Biofilms were formed and treated by *M. alternifolia* as described above with a minor modification: biofilms were formed on polystyrene discs approximately 8 mm in diameter that had been previously sterilized in a 20 -kGy gamma radiation chamber (cobalt 60) for 6 h (Embrarad, São Paulo, Brazil). The discs were placed into 24-well plates (Costa Corning, New York, USA) in which the total volume of suspension, PBS, broth culture and essential oil was 1 mL. The discs were transferred after biofilm formation to 24-well plates fixed in 2.5% glutaraldehyde for 1 h and dehydrated in several ethanol washes (10, 25, 50, 75 and 90% for 20 min each and 100% for 60 min). The plates were then incubated at 37°C for 24 h to dry the discs. The discs were transferred to aluminium stubs and covered with gold for 120 s at 40 mA (BAL-TEC 50D 050 Sputter Coater, Liechtenstein). After metallization, the biofilms were examined and photographed by SEM (Jeol JSM 5600, Tokyo, Japan) operating at 15 kV in increments of 1000 and 5000 times.

### *In vivo*activity of *M. alternifolia*essential oil in oral candidiasis induced in an immunosuppressed mouse model

All animal experiments were conducted in accordance with the policies for animal care, welfare, and use of the Institute of Science and Technology/UNESP and to the Declaration of Helsinki. This study was approved by the Research Ethics Committee of the Institute of Science and Technology/UNESP (protocol number 024/2009-PA/CEP). Twenty-four adult male mice (*Mus musculus*, Albinus, Swiss) with twelve-week-old that tested negative for the *Candida* genus in the oral cavity, weighing approximately 30 to 60 g, were included in the study. Animals were divided into 2 groups: treated with TTO (n = 12) and treated with physiological solution (n =12). In each group, 10 mice were used for analysis by optical microscopy and 2 mice were used for scanning electron microscopy. The design of the experiments is shown in Table 1.

**Table 1:** **Design of the study of** ***in vivo*** **activity of** ***M. alternifolia*** **essential oil**

Day of Experiment	Methodology
Day 1	1° injection of prednisolone
Day 2	Inoculation of *C. albicans* in the oral cavity of mice
Day 5	2° injection of prednisolone
Day 6	Treatment of oral candidiasis with *M. alternifolia* essential oil or physiological solution (control group)
	Recovery of *C. albicans* from the tongue dorsum of mice before and immediately after experimental treatment
Day 7	Euthanasia of the mice: macroscopic analysis, optical microscopy, and Scanning Electron Microscopy (SEM) of the tongue dorsum of mice.

### Induction of experimental candidiasis

The methodology described by Takakura *et al.* [24] was used to induce experimental candidiasis with some modifications. Briefly, animals were immunosuppressed with 2 subcutaneous injections of prednisolone (Depo-Medrol, laboratórios Pfizer Ltda., Guarulhos, SP, Brazil) at a dose of 100 mg/kg of body weight 1 day before and 3 days after infection with *Candida*. Tetracycline chloride (Terramicina, Laboratórios Pfizer Ltda., Guarulhos, SP, Brazil) was administered in the drinking water at a concentration of 0.83 mg/mL beginning 1 day before infection and was maintained throughout the experiment. A 50 μL intramuscular injection of chlorpromazine chloride (10 mg/kg of body weight; Amplictil, Sanofi Aventis, Suzano, SP, Brazil) in each thigh was used to sedate the animals.

*C. albicans* grown for 24 h at 37°C on Sabouraud dextrose agar (Himedia, Mumbai, Maharashtra, India) was resuspended in 10 mL PBS and centrifuged at 358x*g* for 10 minutes. The pellet was resuspended in 10 mL PBS and adjusted to 10^8^ cells/mL after counting in a Neubauer chamber (Laboroptik GMBH, Bad Homburg, Germany). A sterile swab (Absorve, Cral, São Paulo, SP, Brazil) soaked in the *C. albicans* suspension was used to inoculate the sedated mice by rubbing the swab for 1 minute on the tongue dorsum.

### Treatment of experimental oral candidiasis with *M. alternifolia*essential oil

At 4 days post *C. albicans* inoculation, animals were anesthetized by intramuscular injection of ketamine (União Química, São Paulo, Brazil) at a concentration of 100 mg/kg of body weight and Xylazine (Produtos Veterinários J. A. Ltda., Patrocínio Paulista, SP, Brazil) at a dose of 10 mg/kg body weight. The minimal biofilm eradication concentration of *M. alternifolia* determined by the *in vitro* assay (12.5%) was used to treat oral candidiasis. *M. alternifolia* was suspended in 1% Tween 80 and pipetted onto the dorsum of the tongue in 50 μL for 3 times in intervals of 10 minutes. The control group received physiological solution in the same volume and with the same frequency.

### Recovery of *C. albicans*from the tongue dorsum of mice

Samples from the tongue dorsum were collected with a mini-swab before and immediately after each experimental treatment, the swab was placed in a test tube containing 0.99 mL PBS and shaken for 1 minute. This solution was estimated to be diluted by a factor of 10^-2^*Candida* from the soaked swab. Serial dilutions were subsequently made, and 0.1 mL of each dilution was plated onto the surface of Sabouraud dextrose agar (Himedia) containing chloramphenicol (Viximicina, São Paulo, SP, Brazil). Dilutions were plated in duplicate and incubated at 37°C for 48 hours. *Candida* colonies were counted on plates exhibiting 30 to 300 colonies to determine colony-forming units (CFU)/mL. Plates with fewer than 30 colonies from the initial 10^-2^ dilution were estimated to contain 10^-1^*Candida* cells.

### Euthanasia of the mice

One day after the experimental treatment, an excessive dose of anesthetic, 100 mg/kg body weight ketamine (União Química Farmacêutica Nacional S/A., Embu-Guaçu, SP, Brazil) and 10 mg/kg body weight xylazine (Produtos Veterinários J. A. Ltda., Patrocínio Paulista, SP, Brazil) was administered to kill the mice, corresponding to 5 days after experimental candidiasis induction. Tongues were removed for macroscopic and microscopic analyses (optical and scanning electron microscopy, respectively).

### Macroscopic analysis of candidiasis on the tongue dorsum of mice

Characteristic lesions of candidiasis on the tongue dorsum were observed using a stereomicroscope (Zeiss, Göttingen, Germany). For quantification of lesions on the tongue dorsum, scores were assigned from 0 to 4: 0, normal; 1, white patches on less than 20% of the surface; 2, white patches covering between 21% and 90% of the surface; 3, white patches on more than 91% of the surface; and 4, thick white patchy pseudomembranes covering more than 91% of the surface [24].

### Optical microscopy of the tongue dorsum of mice

For microscopic analysis of the lesions, the tongues were fixed in 10% formalin for 24 h. After embedding in paraffin, 5 μm-thick tissue slices were cut and stained with hematoxylin-eosin (H&E) and periodic acid-Schiff (PAS). The presence of candidiasis was analyzed using optical microscopy (Olympus, CX41, Toquio, Japan) at X400 magnification.

Candidiasis lesions were quantified by counting the number of hyphae and epithelial lesions in histological sections stained with PAS and H&E, respectively. For each stain, two histological sections were selected randomly and analyzed from each animal. In each histological section, 21 histologic fields were analyzed in the anteroposterior direction in 42 histologic fields.

The presence of yeasts and hyphae was quantified according to the methodology of Junqueira *et al.* [25], attributing the following scores to histologic fields: 1, 1 to 5 yeasts/hyphae; 2, 6 to 15 yeasts/hyphae; 3, 16 to 50 yeasts/hyphae; and 4, more than 50 yeasts/hyphae. For statistical analysis, a median of the scores obtained from the 42 histologic fields was determined per animal.

The intensity of the tissue lesions was evaluated by counting the number of histologic fields with the presence of epithelial lesions, such as epithelial hyperplasia, disorganization of the basal cell layer, exocytosis, spongiosis, loss of filiform papillae, hyperkeratosis and development of intraepithelial microabscesses. The mean of the number of histologic sections with epithelial lesions was determined per animal for statistical analysis.

### Scanning electron microscopy (SEM) of the tongue dorsum of mice

For SEM analysis, tongues were fixed in 2.5% glutaraldehyde in phosphate buffer (0.1 mol/L and pH 7.3) for 24 h at 4°C. The tongues were washed with a physiological salt solution (0.85% NaCl) for 30 minutes. Specimens were subsequently dehydrated in a series of ethanol solutions (50%, 70%, and 90% for 20 minutes each and 100% for 20 minutes 3 times). After dehydration, tongues were dried to the critical point using CO_2_ (Denton Vacuum DCP 1, Moorestown, NJ). The tongues were then fixed on aluminum stubs and coated with gold for 120 seconds at 40 mA (BAL-TEC 50 D 050 Sputter Coater, Liechtenstein) and evaluated using SEM (JEOL JSM 5600, Tóquio, Japan) at 15 kV. The images obtained from SEM analyses were evaluated only to identify yeasts, hyphae and tissue damage that characterize the experimental candidiasis induced in immunosuppressed mouse model. No quantification of *Candida* or epithelial lesions was performed.

### Statistical analysis of the data obtained from experimental candidiasis

The data obtained from the recovered CFU/mL and quantification of epithelial lesions using optical microscopy were analyzed by Student’s *t* test. The scores from the macroscopic analysis and the quantification of yeasts and hyphae in optical microscopy were evaluated using the non-parametric Mann-Whitney test. A *P* value less than 0.05 was considered statistically significant.

## Results

### *In vitro*activity of *M. alternifolia*essential oil

**Figure 1:**
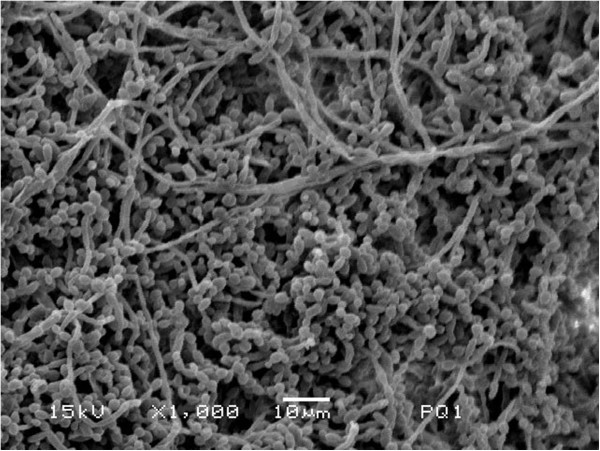
**Scanning electron microscopy (SEM) of** ***C. albicans*** **biofilms formed** ***in vitro*** **on the polystyrene discs.** The biofilm formed after 48 h was composed of blastoconidia, pseudohyphae and hyphae (Control group).

The effects of *M. alternifolia* on the *in vitro* growth of *C. albicans* were first examined following the CLSI. Among the tested concentrations of *M. alternifolia* (0.01 to 25%), the MIC value was determined to be 0.195% (1.95 mg/mL). After determination of the MIC, concentrations of *M. alternifolia* ranging from 0.195 to 25% were tested on *C. albicans* biofilms, and 12.5% was determined to be the minimal biofilm erradication concentration. To confirm complete inhibition of *C. albicans* biofilms after treatment with 12.5% *M. alternifolia*, the biofilms were analyzed by scanning electron microscopy (SEM). In SEM analysis, no formation of hyphae or blastoconidia was observed in the biofilms treated with *M. alternifolia*. However, a heterogeneous biofilm composed of blastoconidia, pseudohyphae and hyphae was observed on the polystyrene discs in the control group without *M. alternifolia* treatment (Figure 1).

### *In vivo*activity of *M. alternifolia*essential oil

**Figure 2:**
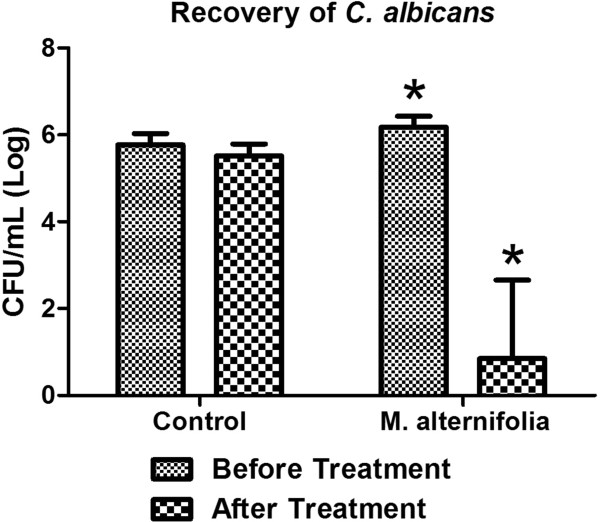
**Number of fungal cells before and after with** ***M. alternifolia*** **treatment.** Means and standard deviations of the CFU/mL of *C. albicans* recovered from the oral cavity of the mice before and after experimental treatment (n = 10 per group). The recovery of *C. albicans* was performed immediately after treatment with *M. alternifolia.* *Significant difference between the number of CFU/mL recovered before and after treatment with TTO (Student’s *t*-test, *P* = 0.001).

The numbers of *C. albicans* recovered from the oral cavity of mice before and immediately after experimental treatments with *M. alternifolia* or physiological solution (control group) are shown in Figure 2. The group treated with *M. alternifolia* showed a 5.33 Log_10_ reduction in the number of *C. albicans* cells after treatment, while the control group had a 0.24 Log_10_ reduction after application of physiological solution, indicating that the essential oil of *M. alternifolia* significantly reduced the colonization by *C. albicans* in the oral cavity of mice.

**Figure 3:**
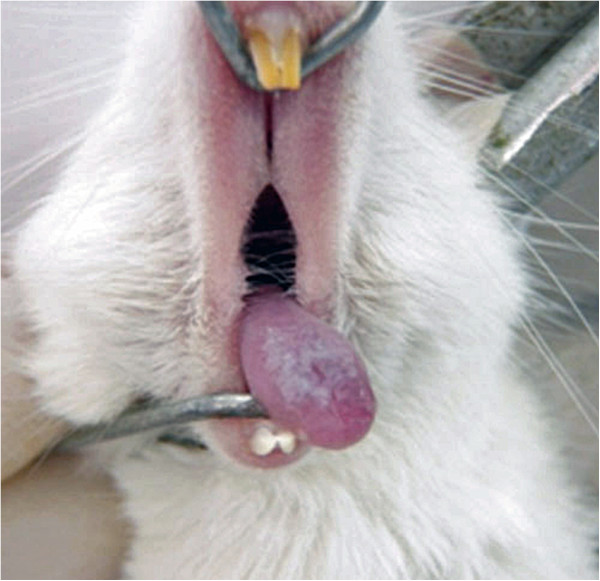
**Macroscopic lesions of candidiasis on the tongue dorsum characterized by pseudomembranous white plaques.**

**Figure 4:**
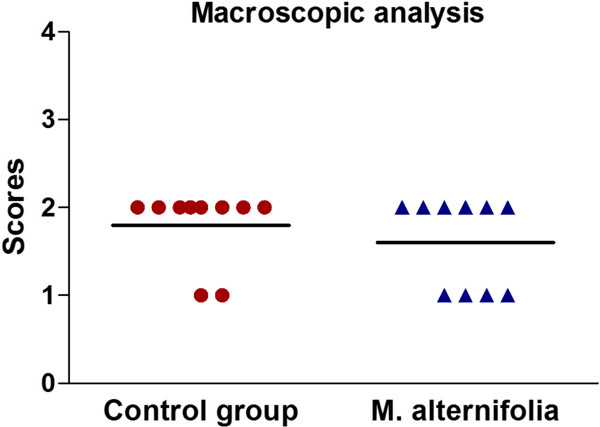
**Macroscopic analysis of candidiasis on the tongue dorsum of mice.** Scores obtained from the macroscopic analysis of candidiasis on the tongue dorsum of mice after 24 h of the treatment with essential oil of *M. alternifolia* or physiological solution for the control group (n = 10 per group). The scores were attributed according to Takakura el al. 2003 as follows: Score 0 (normal), Score 1 (white patches on less than 20% of the surface), Score 2 (white patches covering between 21% and 90% of the surface), Score 3 (white patches on more than 91% of the surface) and Score 4 (thick white patchy pseudomembranes covering more than 91% of the surface). There were no significant differences between the groups treated with *M. alternifolia* oil and control (Mann-Whitney test, *P* = 0.3662).

After 24 h of experimental treatments, macroscopic analysis of the tongue dorsum showed the presence of candidiasis lesions characterized by pseudomembranous white plaques (Figure 3) in both experimental groups (treated with *M. alternifolia* and control) with a median score of 2, representing white patches covering between 21% and 90% of the tongue surface. In all mice studied, only scores 1 and 2 were identified. Although the number of animals receiving score 2 in the group treated with *M. alternifolia* was lower when compared to the control group, this difference was not significant (Figure 4). These data demonstrated that candidiasis lesions persisted for 24 h after treatment with essential oil of *M. alternifolia*.

**Figure 5:**
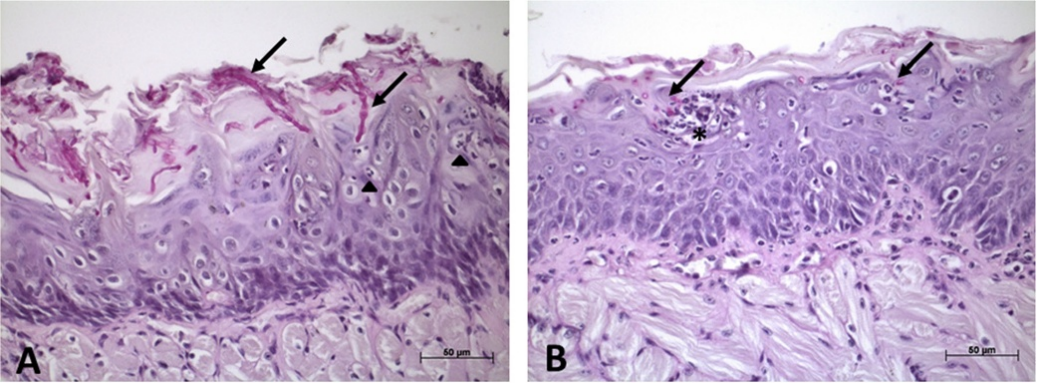
**Sagittal section of the dorsum of the tongue of mice. A)** Control group - Hyphae of *C. albicans* in the keratin layer (arrows) and areas of exocytosis (arrow head). **B)** *M. alternifolia* group - Yeasts of *C. albicans* in the keratin layer (arrows) and intraepithelial microabscesses (*).PAS staining, 400X magnification.

In optical microscopy analysis, candidiasis lesions were represented by the presence of yeasts and hyphae limited to the keratinized layer on the tongue dorsum (Figure 5). In these regions, the epithelial tissue showed loss of filiform papillae, microabscesses, exocytosis, spongiosis, basal layer disorganization, epithelial hyperplasia, and an increased number of mitoses in the basal layer. Muscle inflammation, inflammatory infiltrate and some congested blood vessels in the lamina propria were also observed.

**Figure 6:**
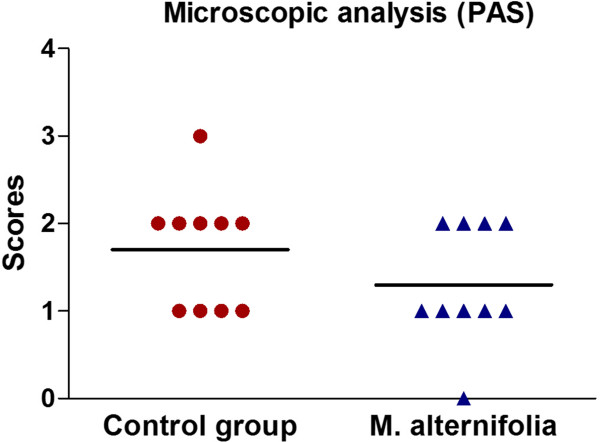
**Microscopic analysis of candidiasis on the tongue dorsum of mice.** Scores obtained from optical microscopy of histological sections stained by PAS after 24 h of the treatment with essential oil of *M. alternifolia* or physiological solution for the control group (n = 10 per group). Scores were attributed according to Junqueira et al. 2005: Score 1 (1 to 5 yeasts/hyphae), Score 2 (6 to 15 yeasts/hyphae), Score 3 (16 to 50 yeasts/hyphae), and Score 4 (more than 50 yeasts/hyphae). There were no significant differences between the groups treated with *M. alternifolia* oil and control (Mann-Whitney test, *P* = 0.2596).

**Figure 7:**
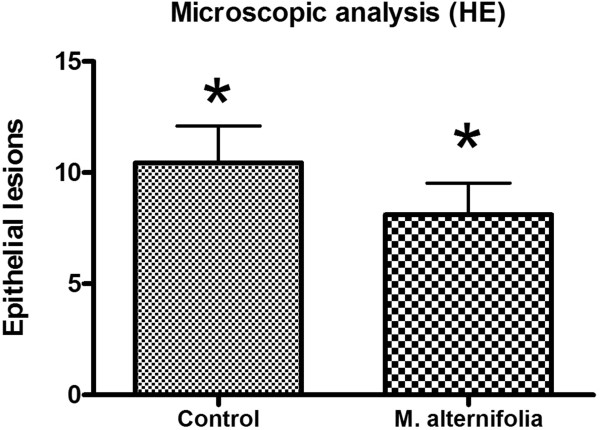
**Means and standard deviations of epithelial lesions obtained from optical microscopy.** Means and standard deviations obtained from optical microscopy of histological sections stained by H&E after 24 h of the treatment with essential oil of *M. alternifolia* or physiological solution for the control group. The number of histological fields with the presence of epithelial lesions was determined for each animal studied (n = 10 per group). *A significant difference between the groups was observed (Student’s *t*-test, *P* = 0.005).

The hyphae and yeasts were quantified in histological sections stained by PAS (Figure 6). Although the median scores obtained for the *M. alternifolia* and control groups were scored 1 and 2, respectively, no statistically significant difference was observed between groups treated with *M. alternifolia* and control (*P* = 0.2596). The epithelial lesions were also quantified using histological sections stained by H&E (Figure 7), and it was observed that the group treated with *M. alternifolia* showed fewer candidiasis lesions when compared to the control group, this difference was statistically significant (*P* = 0.005).

**Figure 8:**
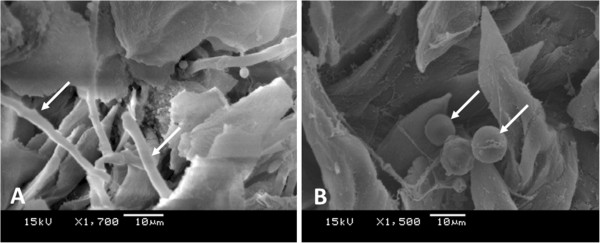
**Scanning electron microscopy of the tongue dorsum. A)** Control group - Hyphae of *C. albicans* (arrows) penetrating the tissue of the tongue dorsum after 24 h of the treatment with physiological solution. **B)** *M. alternifolia* group -Yeasts of *C. albicans* (arrows) on the tissue of the tongue dorsum after 24 h of the treatment with *M. alternifolia oil*.

SEM analysis on the tongue dorsum showed a large quantity of bacteria, yeasts and hyphae. Yeasts appeared in less quantity than hyphae and some hyphae were penetrating the epithelial tissue (Figure 8). In some regions, the filiform papillae were damaged, while papillae were lost in others, showing that the animals developed well-established candidiasis lesions on the tongue dorsum.

## Discussion

Numerous essential oils such as *M. alternifolia* oil have been tested for both their *in vitro* and *in vivo* antifungal activity. *M. alternifolia* has been traditionally used by Australian natives and more recently, it has become a popular essential oil used as a non-ethnic remedy worldwide [12, 20]. In this study, we demonstrated that the essential oil of *M. alternifolia* at a 12.5% concentration completely inhibited the *C. albicans* biofilms formed *in vitro* and had a protective effect against oral *C. albicans* infections in mice.

*M. alternifolia* has demonstrated activity against *C. albicans* [17–20]. New studies that can identify and evaluate the clinical effectiveness of *M. alternifolia* need be developed, such as assays with different experimental models and cell lines by evaluating the cytotoxicity, various concentrations and combinations with conventional antifungal agents to verify the possible synergism. Our findings can contribute in the future to prove *M. alternifolia* as an adjuvant clinical therapy, especially in cases of *Candida* lesions that is the most frequent fungal condition among humans living with HIV.

First, the *in vitro* effects of *M. alternifolia* on *C. albicans* were examined by the broth microdilution method (CLSI), and the MIC obtained was 0.195% (1.95 mg/mL). According to Carson *et al.* [13], the MICs of *M. alternifolia* for yeasts generally range between 0.03 and 0.5%, while fungicidal concentrations generally range from 0.12 to 2%. In addition to inhibiting the growth of *Candida*, some studies have shown that *M. alternifolia* inhibits the formation of germ tubes and decreased the cell surface hydrophobicity of *C. albicans* [13, 17, 18]. Hammer *et al.* [18] verified that germ tube formation was affected by the presence of sub-inhibitory concentrations of *M. alternifolia* oil, and it was completely inhibited by the presence of 0.25% TTO. Sudjana *et al.* [17] investigated the effects of the oil from *M. alternifolia* on *C. albicans* cell adhesion to both abiotic and biotic surfaces. Adhesion of *C. albicans* to polystyrene was significantly reduced for 3 isolates at 0.031%, 6 isolates at 0.062% and 0.125% and for all 7 isolates studied at 0.25% *M. alternifolia*. Similarly, adhesion to buccal epithelial was also significantly reduced in the presence of 0.016-0.062% *M. alternifolia*.

Essential oil mouthwashes containing a range of natural plant extracts, including thymol, eucalyptol, bioflavanoids and *M. alternifolia* oil derivatives, demonstrated direct bactericidal and anticandidal activity *in vitro*. It is thought that essential oil mouthwashes kill microorganisms by cell membrane disruption and enzyme inhibition. However, the effectiveness of natural antimicrobials on established biofilms in the oral cavity is unknown, with incomplete penetration by the agents being reported. The clinical efficacy of essential oil mouthwashes has been studied, but largely against plaque bacteria. Therefore, the clinical benefits of these agents in treating oral candidiasis remain to be established [1].

In this study, we determined the minimal concentration of *M. alternifolia* oil necessary to eradicate *C. albicans* biofilms formed *in vitro*. The minimal biofilm eradication concentration (MBEC) was 12.5%, equivalent to 64 times the MIC. Budzynska *et al.* [11] also evaluated the minimal inhibitory concentration (MIC) and the minimal biofilm eradication concentration (MBEC) of *M. alternifolia* in biofilms formed by *Staphylococcus aureus*. The MIC value was 0.38%, and the authors achieved an MBEC concentration of 173 times the MIC for 1 h of treatment, whereas 8 times the MIC was sufficient to obtain a 90% reduction in biomass metabolic activity after 4 h of treatment.

After establishing the MBEC, we tested the *in vivo* effects of *M. alternifolia* at a 12.5% concentration on oral candidiasis. For this, we used an experimental model of oral candidiasis in immunosuppressed mice developed by Takakura *et al.* [24]; this model is an established methodology that is widely used by several authors for the study of this infection [20, 26–29]. In the control group of this study, a large number of viable *Candida* cells (5-6 Log_10_) were recovered from the oral cavity of mice*.* This finding is in agreement with studies by Costa *et al.* [28] and Ninomiya *et al.* [20] that recovered 5-6 Log_10_*Candida* cells from the oral cavity of animals.

In contrast, a relatively low number of *C. albicans* cells was recovered from the oral cavity of mice treated with *M. alternifolia*. The treatment with this essential oil resulted in a 5.33 Log_10_ reduction of *C. albicans*, indicating clinical efficacy of *M. alternifolia* for the treatment of oral candidiasis. Ninomiya et al. [20] also studied the effects of *M. alternifolia* on oral candidiasis in an immunosuppressed mouse model and found an approximate 1 Log_10_ reduction of *C. albicans* after treatment with *M. alternifolia* at concentrations of 1 and 4%. Probably, the highest *Candida* reduction observed in our study was achieved by the concentration of *M. alternifolia* tested (12.5%).

According to Hammer et al. [30], while the antimicrobial properties of *M. alternifolia* oil are increasingly well-characterized, relatively limited data are available on the safety and toxicity of this oil. Greay *et al.* [31] showed that topical treatments of 10% *M. alternifolia* had significant activity against subcutaneous tumours in immunocompetent mice. The antitumor effect of topical *M. alternifolia* was accompanied by skin irritation similar to other topical chemotherapeutic agents, but unlike other approved topical agents, this irritation was quickly resolved. Furthermore, topical *M. alternifolia* caused an influx of neutrophils in the treated area, with no evidence of systemic toxicity. The lack of systemic activity within the treatment period was verified by normal body weigth, no changes in serum alkaline phosphatase or aspartate aminotransferase and normal liver histology of the animals studied.

Clinical evidence suggests that topical use of the oil is relatively safe and that adverse events are minor, self-limiting and occasional [30]. In a study conducted by Veien *et al.* [32], 217 patients consecutively sampled in a dermatology clinic were patch-tested with 10% *M. alternifolia* with no irritant reactions recorded. Catalán *et al.* [33] tested 20% *M. alternifolia* to identify the *in vitro* and *in vivo* activity of this oil mixed with different tissue conditioners on the *C. albicans* strain. In the *in vitro* study, these authors verified that Coe-Comfort or Fitt conditioners mixed with *M. alternifolia* exhibited total inhibition of *C. albicans*. Patients treated with *M. alternifolia* mixed with Coe-Comfort in an *in vivo* study showed a significant decrease in palatal inflammation when compared to those treated with Coe-Comfort alone. However, *M. alternifolia* can be toxic if ingested, as evidenced by studies with experimental animals and from cases of human poisoning [34, 35]. The 50% lethal dose for *M. alternifolia* in a rat model is 1.9 to 2.6 mL/Kg. Incidences of oral poisoning in children and adults have been reported, and in all cases, patients responded to supportive care and recovered without apparent sequelae [13].

In this study, *M. alternifolia* was suspended in 1% Tween 80 to be applied on the dorsum of the tongue. Tween 80, a nonionic surfactant and an emulsifier, is frequently used as a carrier for *M. alternifolia*. Considering the biological effects of Tween 80 in experimental oral candidiasis treated by *M. alternifolia*, Ninomiya *et al*. [20, 36] used 1% Tween 80 as control group and found similar results of *C. albicans* CFU/mL compared to other studies of oral candidiasis in immunosuppressed mice that used physiological solution as control group. Several studies showed that Tween 80 has low toxicity for human cells and experimental animals [37, 38]. In addition, its application is permitted for intradermal and intravenous injection in human beings [38].

After verifying that *M. alternifolia* at concentration of 12.5% significantly reduced the *C. albicans* colonization in the oral cavity of mice, we tested the effects of *M. alternifolia* treatment on the candidiasis lesions and tissue repair. Therefore, mice were killed 24 hours after the treatment and the tongues were removed for macroscopic and microscopic analyses. In these analyses, we verified that the animals treated with *M. alternifolia* showed fewer candidiasis lesions when compared to untreated animals (control group). Despite this, no statistically significant difference was observed for the macroscopic analysis and quantification of hyphae (histological sections stained by PAS) between *M. alternifolia* and control-treated groups. Only the quantification of epithelial lesions (histological sections stained by H&E) showed a statistically significant difference between groups.

However, Ninomiya *et al.* [20] found a significant reduction in the macroscopic lesions of the dorsum tongue for groups treated with *M. alternifolia*. This difference might be attributed to the time of *M. alternifolia* application in relation to the stage of candidiasis infection. Ninomiya *et al.* [20] applied *M. alternifolia* on the dorsum tongue at 3 and 24 h after infection by *C. albicans*. Because it is known that *C. albicans* form germ tubes and hyphae 3 hours after inoculation in experimental oral candidiasis [39], Ninomiya *et al.* [20] may have found a significant reduction in macroscopic lesions because *M. alternifolia* prevented *Candida* invasion of the epithelium by hyphae. These data suggest that early administration of *M. alternifolia* is highly effective. In the present study, *M. alternifolia* was applied 4 days after infection by *C. albicans*. Therefore, oral candidiasis lesions were already well-established, and consequently impaired the action of *M. alternifolia*. Furthermore, we evaluated candidiasis lesions on the dorsum tongue 24 h after application of *M. alternifolia*, and this time period may not have been long enough for improvement of the lesions and tissue repair to occur.

## Conclusions

In this study, we concluded that *M. alternifolia* oil at a 12.5% concentration was effective to eradicate a *C. albicans* biofilm formed *in vitro* and to reduce yeasts of *C. albicans* in an immunosuppressed mouse model.
